# Entropy-Based Approach for the Detection of Changes in Arabic Newspapers’ Content

**DOI:** 10.3390/e22040441

**Published:** 2020-04-14

**Authors:** Olga Bernikova, Oleg Granichin, Dan Lemberg, Oleg Redkin, Zeev Volkovich

**Affiliations:** 1 Research Laboratory for Analysis and Modeling of Social Processes, Saint Petersburg State University, Universitetskaya nab. 7-9, Saint Petersburg 190000, Russia; o.bernikova@spbu.ru (O.B.); o.redkin@spbu.ru (O.R.); 2 Faculty of Mathematics and Mechanics, and Research Laboratory for Analysis and Modeling of Social Processes, Saint Petersburg State University, Universitetsky prospekt 28, Saint Petersburg 198504, Russia; o.granichin@spbu.ru; 3 Software Engineering Department, ORT Braude College of Engineering, Karmiel 21982, Israel; lembergdan@braude.ac.il

**Keywords:** publishing model modeling, anomaly detection, word embedding

## Abstract

A new method for the recognition of meaningful changes in social state based on transformations of the linguistic content in Arabic newspapers is suggested. The detected alterations of the linguistic material in Arabic newspapers play an indicator role. The currently proposed approach acts in an “online” fashion and uses pre-trained vector representations of Arabic words. After a pre-processing stage, the words in the issues’ texts are substituted by vectors obtained within a word embedding methodology. The approach typifies the consistent linguistic template by the similarity of the embedded vectors. A change in the distributions of the issue-grounded samples indicates a difference in the underlying newspaper template. A two-step procedure implements the concept, where the first step compares the similarity distribution of the current issue versus the union of ones corresponding to several of its predecessors. A repeating under-sampling approach accompanied by a two-sample test stabilizes the sampling and returns a collection of the resultant *p*-values. In the second stage, the entropy of these sets is sequentially calculated, such that the change points of the time series obtained in this way indicate the changes in the newspaper content. Numerical experiments provided on the following issues of several Arabic newspapers published in the Arab Spring period demonstrate the high reliability of the method.

## 1. Introduction

Currently, the mass media have a powerful influence on modern society due to their close association with the so-called “mediated culture”. This modern phenomenon exposes people’s opinions and expectations through a vast quantity of documents created in social networks and other discussion forums. Monitoring and comprehending the deliberated topics is an essential element of each trustworthy modern multimedia system. Therefore, the study of social network chatting is a useful forecasting tool in many areas, such as election results [1,2], criminal activity [3,4], and social events [5,6,7]. This kind of analysis sensibly summarizes the feelings and aims revealed in social networks without any relation to texts’ linguistic content, while it is generally an outcome of the collective creativity uncovered by an informal language in a faithful matter.

On the other hand, the old-style media (television, radio, and newspapers) are one-way information systems conveying most of the material by a sufficiently official language. Systems of this kind (whether written, broadcasts, or conversations) predictably track the day’s occasions and often express their points of view. Newspapers, being the de facto source of all traditional media, are bundles of connected articles with content gathered by a small group of people frequently sharing a common bias caused by changes in political, economic, and social events in society and dominant elites and exploiting them as propaganda resources.

Propagandistic purposes can be achieved by means of a language adaption of electronic and print media following the characteristics of the target audience taking into account the level of education, political preferences, customs and traditions, gender, and linguistic (dialectical) features. For these reasons, it is natural to suppose that changes in the media language content may expose changes in social status. As was mentioned in [8] “The lack of a genuine will to liberate state media in the so called Arab Spring countries from the grip of political power was, and is still a major handicap. In the immediate aftermath of the uprisings, state media journalists managed to challenge the reverential, uniform style and content they’d had to adopt for decades, but only very briefly. They quickly reverted to old practices in the face of raging political battles and attempts by current regimes to again use state media as a means to justify and legitimize their policies.”

Media in English is an essential object of study. However, at least 422 million people in 57 countries use Arabic as their primary language and 250 million as their second one. Indeed, Arabic is the fifth amongst the most widely spoken conversational tongues across the world. Arabic is more complicated language grammatically and morphologically in comparison with the European ones. Analysis of the traditional Arabic media attracts attention from this standpoint and the aspiration to study the conventional Arabic press to reflect the government’s opinions and expectations.

In this article, a new method for online recognition of meaningful changes in social status is suggested. The detected transformations of the linguistic content in Arabic newspapers play an indicator role. The possibility of such modeling is predominantly proven in [9]. The mentioned methodology represents each issue as a frequency vector of appropriately chosen character *N*-grams, so that the writing style evolution of a newspaper is depicted by employing a mean correlation between the current issue and several of its forerunners. Change points of the constructed in this way time series indicate possible changes in the linguistic content. However, the obtained data sequence is quite noisy and requires further smoothing, which is done via a wavelet transform and a clustering procedure across all given issuing periods. Thus, this process involves information about “the future published issues”.

The currently proposed approach acts in an “online” fashion. After a pre-processing stage, the words in the issues’ texts are exchanged by vectors obtained within a word embedding methodology. The collection of all similarities (in our application, the well-known “cos-similarities”) of the appropriately chosen adjacent vectors typifies the consistent linguistic template of the issue, and a change in distributions of these issue-based samples indicates a difference in the underlying newspaper template.

To implement this concept, a two-step procedure is proposed. The first step consists of evaluating the current issue sample distribution versus the union of ones corresponding to several issue’s predecessors. We use, aiming to stabilize this inherently imbalanced procedure, a repeating under-sampling approach accompanied by a two-sample test. In the second stage, the normalized entropy of the returned *p*-values is calculated to form a time series of the newspaper’s representation. Notice that in the case of the stable style behavior, the entropy fluctuates almost to its maximum value, and each significant descent is probably due to a difference in the language content of the issue and its predecessors.

The rest of the paper is organized in the following way. Section 2 is devoted to the necessary background. Section 3 presents the novel evolutionary model of the publishing process. Section 4 is devoted to the experimental study results. Section 5 consists of discussion and conclusions.

## 2. Background Knowledge

Many traditional techniques in the text mining area are associated with vector representations (e.g., bag-of-words) of a text as vectors of terms’ occurrences. It is a well-known fact that this methodology loses semantic information about words’ sequencing, namely disregarding the order of words and their mutual appearances. Another common Markov *N*-gram approach takes into account the word arrangement in short sentences, but leads to sparse representations. Deep learning embedding systems provide innovative tactics for the treatment of the sparsity problem by supplying words’ representation as real-valued vectors such that close vectors present words with comparable sense. The conceptual leap involves the idea to connect each attribute, not to a single dimension, but to a whole dense vector. It makes it possible to translate a given text to a matrix with semantically taught columns. Hence, word embedding is known as a suite of language modeling methods presenting words of a given glossary in digital vector spaces, usually having high dimensionality. This incredibly valuable technique for natural language processing preserves the essential semantic and syntactic information of terms allowing adjusting the performance in many natural language processing tasks. Popular off-the-shelf word embedding models in use today are:

- Word2Vec (by Google) [10]
- GloVe(by Stanford) [11]
- FastText (by Facebook) [12]
- ELMo(AllenNLP’s) [13]

The purpose of embedding is to grant close spatial positions to words with a comparable context. Formally, the “cos-similarity”, calculated as the angle between such vectors, has to be close to one. The selection of the dimensionality, commonly denoted as *d*, of the constructed vectorial representation, is a famous open problem connected to choosing an appropriate sub-optimal solution. Apparently, d=300 is the most frequently used value.

Hypothesis testing (see, e.g., [14]) is another tool involved in the model. This methodology is one of frequently employed in various branches of machine learning and data mining due to its numerous successful applications (see, e.g., [15]). Two-sample hypothesis testing is an approach invented to examine if two samples of independent random values have the same probability distribution function. In this case, the null and alternative hypotheses are formulated such that the null hypothesis $\left(H_{0}\right)$ reveals the “no difference” assumption, and the alternative hypothesis $\left(H_{1}\right)$ claims that there is “a difference in the underlying distributions”. Formally speaking, let $X=X_{1},X_{2},\ldots ,X_{m}$ and $Y=Y_{1},Y_{2},\ldots ,Y_{n}$ be two independent random variables with unknown distribution functions *F* and *G*. A two-sample problem tests the null hypothesis:

$$H_{0}:F\left(x\right)=G\left(x\right)$$

against the alternative:

$$H_{1}:F\left(x\right)\neq G\left(x\right).$$

A test is typically made using a statistic calculated from drawn samples together with its *p*-value, which is the probability of the actual instances happening, if the null hypothesis is correct. Therefore, the *p*-value can be understood as a random variable generated from the drawn samples. The standard application of the probability integral transform of the test statistic based on the null hypothesis distribution allows concluding that the scattering of the calculated *p*-value is uniform in [0,1] if the null hypothesis is correct.

Supposedly, the most useful and general two-sample test is the Kolmogorov–Smirnov one (the KS-test) [16,17]. This nonparametric procedure works with the continuous, one-dimensional probability distributions and uses the statistic:

$$D=\underset{x}{\sup}\left|\tilde{F}\left(x\right)-\tilde{G}\left(x\right)\right|$$

evaluating the distance between the empirical distribution functions $\tilde{F}\left(x\right)$ and $\tilde{G}\left(x\right)$ of the samples. For sufficiently large samples, the distribution of the presented statistic does not depend on the underlying distributions of the data.

## 3. Evolutionary Model of the Publishing Process

This section presents a dynamic model of the daily publishing process based on the paradigm of the human writing process. A shared outlook (see, for example, [18]) of this development calls up four central parts: planning, drafting, editing, and writing the final draft. Consequently, this perception naturally suggests the inherent linguistic and semantic dependence of the current text segment with formerly written ones. This presumption is also, to some extent, correct for sequential newspaper issues. Nevertheless, a book’s content appears to be much smoother since it passes an iterative editing exercise commonly less feasible for daily media. The regular publishing process is more like an industrial system functioning with the influence of the opinions of a group of authors and their writing styles.

The proposed model handles a time series constructed to reflect the semantic comportment of dailies. In our stance, anomalies surfacing in such a series are mainly caused by significant changes in the social state. Let us consider a series of *m* sequential issues:

(1) $$D=\{D_{1},D_{2},\ldots ,D_{m}\}$$

of a newspaper and take a vocabulary of terms V with an embedding matrix $\mathrm{Emb}\in R^{\left|V\right|\times d}$ associating the words from the vocabulary V with *d*-dimensional vectors, where the columns correspond to the word embedding. The semantic evolution of D is modeled in our approach as follows.

1. Embedding step: At the outset, each word w∈V in all issues is transformed into a *d*-dimensional vector according to the appropriate column in the matrix Emb. Subsequently, each issue $D_{i},\;i=1,\ldots ,m$ is converted to a two-dimensional array $M_{i},\;i=1,\ldots ,m$ by sequentially concatenating the corresponding vector representations of its words.
2. Semantic issues’ pattern: As was mentioned, the intention of a word embedding procedure is to convert words with similar contexts to have close spatial shapes. A similarity measure between real-valued vectors (like cosine or the Euclidean distance) provides an accepted tool to quantify the words’ semantical relationship. As an issue is composed of words, the similarity between words can typify its semantical structure. The Euclidean distance takes into account a vector magnitude, while the cosine similarity depends on just the angle between the vectors. Therefore, this measure is more robust to changes in the frequencies of the semantically similar word, whereas the magnitude is sensible to occurrences and neighborhood diversity. For this reason, the resulting vectors of “semantically similar” terms produced, for example, by the Word2vec procedure may be close in cosine similarity, but still have a sizeable Euclidean distance between them.
  From this point of view, the cos-similarity between adjacent words can represent the desired semantic configuration. Keeping it up, each issue $D_{i},\;i=1,\ldots ,m$ is represented by its pattern distribution $G_{i,l,h},\;i=1,\ldots ,m$ being a distribution of the cos-similarities calculated within a window with size *l* sliding in the increment of *h* over the columns of the corresponding matrix $M_{i},\;i=1,\ldots ,m$.
  Semantic change model: The collection of the pattern distributions $G_{i,l,h}$ of the cos-similarities found for neighboring terms represents the daily under consideration. For a given issue $D_{i}$, introduce:
  $$G_{T,i,l,h}=\overset{i-1}{\underset{j=i-T}{⋃}}G_{j,l,h},$$
  where *T* denotes the number of $D_{i}$ several “precursors” involved in the assessment. According to the model under consideration, a semantic change does not occur if two collections $G_{T,i,l,h}$ and $G_{i,l,h}$ are identically distributed, i.e., the connection between contiguous words is kept. Thus, the model deals with the classical hypothesis testing to determine if two underlying distributions are equal. An essential tool to verify this is a two-sample test intended to check if two samples are drawn from the same population. However, we compare two substantially differently-sized sets. Obviously, the size of $G_{T,i,l,h}$ is expected to be approximately *T* times larger than $G_{j,l,h}$. To overcome this problem, the multiple testing metrology can be applied. Specifically, let us select a natural number *N* and a two-sample test *H* returning within its output parameters the resulting *p*-value. A multiple-test procedure consists of the following steps presented in Algorithm 1.
**Algorithm 1** Constructing representative sets:*Input:*$G_{i,l,h},G_{T,i,l,h}$*: two sets to be tested*$\left(\left|G_{T,i,l,h}\right|\geq \left|G_{i,l,h}\right|\right)$*;*H: two-sample test;Nsize*: sample size;*N: number of samples drawn.*Procedure:*counter=0*Repeat N times:*(a)   counter=counter+1(b)   *Draw random sample Stem p without replacement from $G_{T,i,l,h}$ with size*Nsize.(c)   *Apply H, and obtain the current p-value*$$p\left(counter\right)=H\left(G_{i,l,h},G_{T,i,l,h}\right)$$*Return the array p*
  Thus, the stylistic relationship between the issue $D_{i}\;\left(i>T\right)$ and its *T* “precursors” $\left\{D_{j},\;j=i-T,\ldots ,j-1\right\}$ is represented as a set:
  $$Z_{i,T,N,V}=\left\{p_{1},\ldots ,p_{N}\right\}$$
  of the calculated *p*-values.
3. Entropic time series: In our approach, the null hypothesis about the equality of the primary distributions of $G_{T,i,l,h}$ and $G_{i,l,h}$ is tested against the alternative hypothesis stating the difference of the distributions. As was mentioned in Section 2, the distribution of the *p*-values found here is uniform in [0,1] if the null hypothesis is true. As a result, the stationary behavior of semantic sets $\left\{Z_{i,T,N,V},\;i=1,\ldots ,m\right\}$ is characterized by their sufficiently high entropy, and a reduction of the entropy value indicates a change in the language content and probably a change in the social state. Consecutive assessment of such entropy values leads to the following time series (one-dimensional signal):
  $$S_{i}=Ent\left(Z_{i,T,N,V}\right)/\log_{2}\left(N\right),\;i=1,\ldots ,m$$
  exposing the semantic evolution of the newspaper. Normalization by the maximal entropy value is provided, aiming to standardize the entropy behavior for different sample sizes. Therefore, the stable signal corresponds to the steady linguistic content of the newspaper, and its acute falling indicate changes in one.
4. Anomaly detection: Anomalous falls of a signal may specify the desired change points, and therefore, an anomaly detection method is an essential tool for this purpose. In this paper, the standard modified Thompson Tau test [19] is applied. The mentioned method is a famous process intended to recognize outliers in a set. The approach supplies a statistically clarified rejection zone to decide if a data point is an outlier resting upon the standard data deviation and the average. This method consists of the following: Let *X* be a vector of size *n*. Denote by $\bar{X}$ the average of *X* and by σ(X) the standard deviation of *X*. The rejection threshold is determined using the formula:
  $$rej=\frac{t_{\alpha /2,n-2}\left(n-1\right)}{\sqrt{n\left(n-2+t_{\alpha /2,n-2}^{2}\right)}},$$
  where $t_{\alpha /2,n-2}$ is the critical value from the Student distribution based on significance level α and degree of freedom n−2. For each data point x∈X, the value is calculated as:
  $$\delta \left(x\right)=\frac{|x-\bar{X}|}{\sigma (X)},$$
  and then, if δ(x)>rej, a data point is recognized as an outlier, else (if δ(x)≤rej) a data point is not considered as an outlier.
  The described procedure is applied in our model in the following manner. Locating at the position *i* corresponding to the current issue $D_{i}$, we construct a sequence $s_{i-L+j},\;j=1,\ldots ,L$ containing L−1 “precursors” of s(i) and this value itself. In the following step, the standard modified Thompson Tau test checks if s(i) is an outlier in the constructed series.

It should be noted that the proposed method, in fact, works in a real-time mode since, at each current moment, it just uses information about previously published issues of a newspaper. The following Algorithm 2 presents such an “online” version.
**Algorithm 2***Input:*V: a vocabulary of terms;$\mathrm{Emb}\in R^{\left|V\right|\times d}$: terms’ embedding matrix;l: size of a sliding window;h: sliding increment;T: number of “precursors” compared with the current issue;H: two-sample test;Nsize: sample size;N: number of resamplings;α: significance level of the modified Thompson Tau test;L: lag parameter in the anomaly testing procedure.*Procedure:*Get the current issue $D_{i},\;i>T_{0}=\max\left(T,L\right)$Construct the embedding matrices $M_{j}$ of $D_{j},\;j=i-T_{0},\ldots ,i$ using the underlying embedding matrix $\mathrm{Emb}\in R^{|V|\times d}$Construct the cos-similarities collections $G_{i,l,h}$ between the columns of matrices $M_{j}\;j=i-T_{0},\ldots ,i$ using sliding window with parameters (l,h)Apply Algorithm 1 with the parameters H,N, and Nsize to the sets $G_{i,l,h},G_{T,i,l,h}$, and get the representative sets $Z_{j,T,N,V},\;j=i-T_{0},\ldots ,i$Construct a time series sequence of the normalized Entropy values: $S_{i}=\left\{S_{j},\;j=i-T_{0},\ldots ,i\right\}$Check using the modified Thompson Tau test if $S_{i}$ is an outlier in $S_{i}$If the next issue of the newspaper is available, then goto 1; otherwise stop


## 4. Numerical Experiments

### 4.1. Material

The Arabic alphabet consists of 28 letters. There is no difference between uppercase and lowercase letters or between written and printed letters. Some letters are connected to adjacent letters in words on both sides, while others are connected only on the right. Since Arabic writing forms a “ligature”, each letter can have up to four different forms, depending on where the letter occurs in the word. This is just one of the many reasons why Arabic has a more complex morphology compared to other languages such as English or Russian. A vital step in the construction and application of any word embedding model, especially in the Arabic language, is text preprocessing because such a procedure has a strong ability to influence the outcomes. The embedding model used in this research is the known AraVecopen-source project [20] intended for the Arabic NLP research community. The technique suggests a preprocessing containing filtering of non-Arabic content and normalization of the Arabic characters involving several actions such as combining and replacing certain characters or words and dialectic removing.

The experiments are provided in an imitation manner, where the publishing process is simulated daily, aiming to recognize noteworthy social events, in issues of the following Arabic newspapers:

- “Al-Ahraam”, (“The Pyramids, Egypt”);
- “Akhbaar Al-Khaleej”, (“The News of the Gulf Bahrain”);
- “Al-Ghad”, (“The Tomorrow Jordan”).

The oldest and the most valued is “Al-Ahraam” (founded in 1875), mainly exposing the official position of the government. “Akhbaar Al-Khaleej” and “Al-Ghad” were established in 1976 and 2004, correspondingly. A Bahraini “Akhbaar Al-Khaleej” newspaper is also pro-governmental, although “Al-Ghad” from Jordan is respected as the first independent Arabic daily. The newspapers vary in the edition: “Al-Ahraam” has a flow of around 900,000; “Akhbaar Al-Khaleej” prints 37,000 copies; and “Al-Ghad” has a distribution of 38,000–42,000 copies.

### 4.2. Parameters’ Selection

For high values of the delay parameter *T*, the resulting entropy curve is expected to be smoother. However, the necessary information could be lost. A balance point between these clashing factors could provide a suitable estimation of *T*. We chose T=20 as such a poise point. The size *l* of the sliding windows has to be sufficiently small since a suitable word association expectedly appears inside adequately narrow intervals. Therefore, we chose l=5 with a stride value h=1. The significance level of the modified Thompson Tau test was is $\alpha_{Th}=0.01$ with the lag L=20. As a two-sample test, we used the Kolmogorov–Smirnov test with Nsize=500. The number of samples *N* is calculated as:

$$N=\frac{\left|G_{T,i,l,h}\right|}{2Nsize}.$$

### 4.3. Results

#### 4.3.1. “Al-Ahraam”

“Al-Ahraam” (Arabic: “The Pyramids”), instituted on 5 August 1875, is the most popular Egyptian daily, being the second oldest after “al-Waqa’i’ al-Masriya” (“The Egyptian Events”, inaugurated in 1828 on the order of Muhammad Ali). It is a well-known daily, not only in the country, but also in the Arabic world as a whole. The studied dataset consists of 909 issues published into periods:

- 1.1.2010–31.12.2011.
- 1.1.2014–30.6.2014.

**Figure 1:**
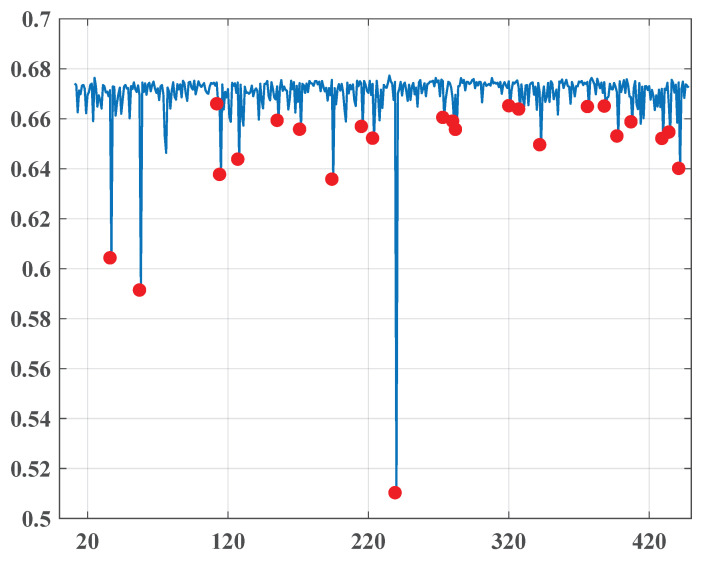
The overall entropy graph of the “Al-Ahraam” newspaper in the first time frame.

**The first time frame:** This period contains 730 issues of the newspaper. The overall entropy graph is given in Figure 1, where the found change points are marked in red.

**Figure 2:**
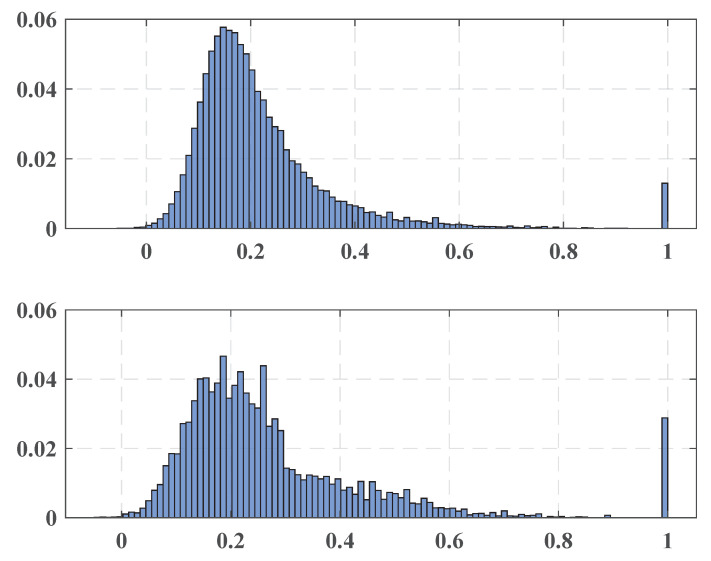
Examples of the similarity distributions.

The following Figure 2 presents two examples of the similarity distributions of a regular issue (top plot) and an issue corresponding to a style change point.

As can be seen, the proportion of word pairs with low similarity is more considerable in the first case, whereas in the second case, the distribution is significantly less concentrated. Thus, it is possible to assume that while a change point occurs, the content of the corresponding issue is more focused on a specific topic. The found change points are presented in the following Table 1.

Let us consider the adjustments in the social state associated with the found change points.

1. October, November-2010
  - October 27, 2010
  - November 17, 2010
  Note that the points are quite far from each other, so their occurrence may not be directly associated with significant social changes, although they are located quite close to the date of the parliamentary elections in Egypt on 28.11.2010 and may correspond to the expectations of possible changes. Demonstrations and uprisings swept across the whole country as citizens complained of suspected fraud in parliamentary elections. The protests were sustained by local and international human rights groups.
2. January-2011
  - January 11, 2011
  - January 13, 2011
  - January 26, 2011
  This quite dense group of change points very well predicts 25.1.2011 (the “Day of Revolt”) of the famous January 25 Revolution, which began on 25.1.2011 and unfolded across Egypt. Abundant, mainly non-violent demonstrations, apparently encouraged by Tunisian street protests, exploded throughout the country, demanding the resignation of President Hosni Mubarak and led to his resignation on the night of 11.2.2011.
3. February, March-2011
  - February 23, 2011
  - March 11, 2011
  The interim Cabinet of Ministers was sworn in on 22.2.2010. This cabinet incorporated the opposition delegates, but Mubarak’s appointees still held some critical positions that caused new demonstrations demanding their removal. The event is visibly connected to the first point set on 23.2.2011.
  The second point is clearly associated with the events that followed. On 3.3.2011 and 7.3.2011, Prime Minister Ahmed Shafiq retained by Mubarak resigned and was replaced by Essam Sharaf, known as a Mubarak’s opponent. Moreover, the new government did not contain persons closely associated with Mubarak.
4. April-2011
  - April 3, 2011
  - April 24, 2011
  On Friday, 1.4.2011, a large number of demonstrators filled Tahrir Square after prayers with the demand to “Save the Revolution” to the governing military council. On 9.4.2011, the army applied force to expel people, killing two and wounding many. Finally, on 13.4.20111, Egypt’s State Prosecutor ordered that Mubarak and his sons Alaa and Gamal be detained for 15 days for questioning.
5. May-2011
  - May 2, 2011
  - May 18, 2011
  On 5.5.2011, former Egyptian Interior Minister Habib al-Adli was sentenced to 12 years in prison for corruption. He was convicted after removing Mubarak from his post and was awaiting trial for allegedly ordering security forces to fire at protestants. On 24.5.2011, Egypt’s Public Prosecutor declared that Mubarak and his sons Alaa and Gamal would stand trial for ordering security forces to shoot protesters and for corruption. Apparently, these dramatic changes in the political status of the elite associated with the former President caused a great change in the pro-government media. This fact is reflected by the sharpest fall of the entropy (0.512) in the whole considered period on 18.5.2011.
6. June-2011
  - June 21, 2011
  - June 28, 2011
  - June 30, 2011
  These change points are precisely associated with a exciting social explosion in the summer of 2011. Thousands of black-dressed Egyptians took part in marches on 6.6.2011 to honor Khaled Saeed. The protests continued so that on 28.6.2011, a bloody clash resulting in numerous victims happened in central Cairo between demonstrators and security forces. Further, the “Friday of Retribution” took place on 1.7.2011 when crowds in Suez, Alexandria, and Tahrir Square in Cairo expressed deep disappointment with the governing of the Supreme Council of the Armed Forces. Similar rallies, named the “Friday of Determination” and the “March of the Million”, happened on 8.7.2011 in Suez, Alexandria, Cairo, and other cities.
7. August-2011
  - August 7, 2011
  - August 21, 2011
  Apparently, each one of the occurred points related to different events. The first one could be associated with the first time Mubarak had a public appearing as his trial commenced in Cairo amid heavy security. Due to a large number of lawyers in court representing the families of slain protesters, the media desperately covered the process that is expressed by the first change point. Eight people were killed on 18.8.2011 in a shooting attack on an Israeli bus near the Egyptian border. During the incident, five Egyptian police officers were also killed by the Israeli side, which caused public outrage in Egypt. As a result, Egypt declared that it would withdraw its ambassador to Israel. Protests happened at the Israeli Embassy in Egypt. The crisis produced by the attack was understood as a signal for worsening relations between the two countries in the post-Mubarak era and reflected by the content change on 21.8.2011.
8. October-2011
  - October 2, 2011
  - October 14, 2011
  - October 23, 2011
  On the evening of 9.10.2011, Coptic Christians mobilized in Cairo to protest the burning of a church in Upper Egypt and to demand the resignation the Supreme Council of the Armed Forces together with the chairman, Field Marshal Mohamed Tantawi, and the superintendent of Aswan province. In the attack undertaken by policy, about 25 people were killed and about 200 injured. Moreover, the army forces also captured “Al-Hurra TV” station and “25 January TV stations”. The State Media was requested to cover these events in a benevolent manner for the military junta. A change points group seemingly appeared as a response to this request.
9. November-2011
  - November 24, 2011
  - November 29, 2011
  The second half of November symbolized the resumption of a harsh confrontation between demonstrators and the authorities in reaction to the military power unilaterally announcing a super-constitution. As a result, on Saturday 19.11.2011, many people suffered during the demonstration crackdown. This notwithstanding it, the Supreme Council asserted that the parliamentary elections would start as planned on 28.11.2011. The cabinet headed by Prime Minister Essam Sharaf walked out as a protest against this decision and the decision to use force against the protesters. An agreement for a new interim government was achieved on the following day 22.11.2011.
10. December-2011
  - December 6, 2011
  It is doubtful that this fairly isolated point reflected a significant change in style. Supposedly, this could be associated with a local style fluctuation as a reaction to the continuing civil protests and the results published on 30.11.2011 of the first round of the parliamentary elections, demonstrating the overwhelming advantage of the Muslim Brotherhood’s Freedom and Justice Party and another hard-line Islamist party, the Nūr Party.

**Figure 3:**
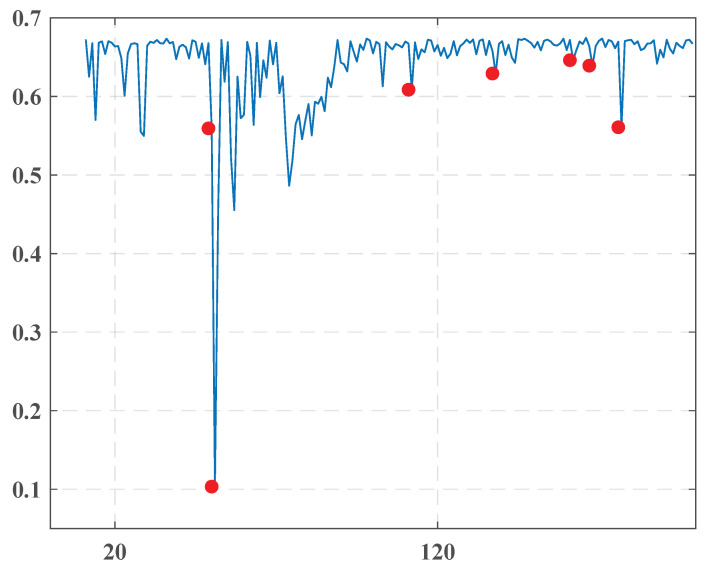
The overall entropy graph of the “Al-Ahraam” newspaper in the second time frame.

**The second time frame:** A problem arises with handling these data consisting of the fact that the main essential events are expected to happen within the starting two of three weeks. The proposed model suggested having an opening training period with a duration of at least the maximum between L and T, where the procedure learned the stable behavior of the system. To overcome this difficulty, the last 30 issues of the first time frame are sequentially inserted into the dataset before the first issue of the second time frame. As was demonstrated earlier, the newspaper performances are almost stable in this period and could serve as training material. The found change points are presented in Figure 3 and Table 2.

Let us look thoroughly at the found change points.

1. January-2014
  - January 11, 2014
  - January 12, 2014
  This dense group with a very sharp drop of the entropy on 12.1.2014 is naturally connected to a constitutional referendum in Egypt held on 14.1.2014 and 15.1.2014, where 98.1% of voters approved the new constitution. The nomination of the new Editor and Editor-in-Chief of “Al-Ahraam” happened on 2.1.2014 completely changed the pro-government coverage of the political situation and really predicted the results of the plebiscite.
2. March-2014
  - March 16, 2014
  Presumably, this isolated change point foresaw a verdict of an Egyptian court, which condemned 529 followers of the Muslim Brotherhood to death on 24.3.2014.
3. April-2014
  - April 11, 2014
  It is difficult to interpret this isolated point with certainty. Perhaps its occurrence could be associated with the violent clashes that took place in Egypt in April 2014. However, there probably is no direct link.
4. May-2014
  - May 5, 2014
  - May 11, 2014
  - May 20, 2014
  These points are undoubtedly related to a presidential election in Egypt in the period from 26.5.2014 to 28.5.2014. Abdel Fattah al-Sisi was voted in with 97% of the electors, consistent with the Egyptian official declaration. The President was sworn into office on 8.6.2014.

#### 4.3.2. “Akhbaar Al-Khaleej ”

**Figure 4:**
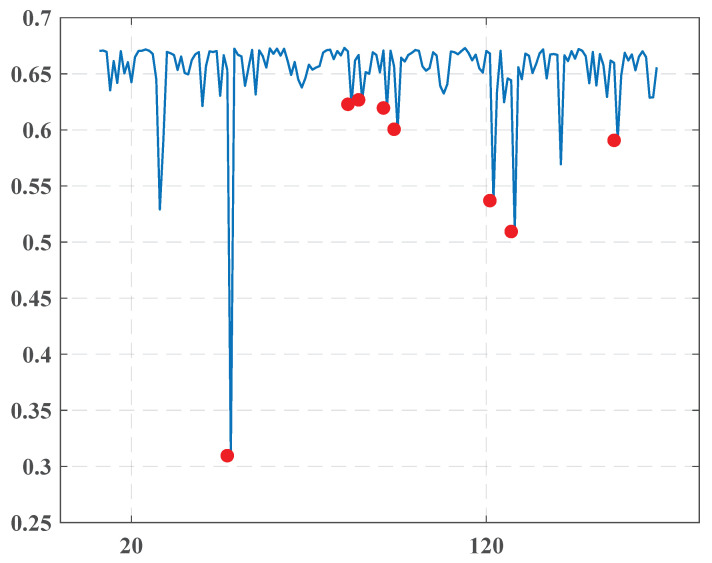
The overall entropy graph of the “Akhbaar Al-Khaleej” newspaper.

The dataset consisted of 178 issues of the newspaper in the interval from 25.12.2010 to 24.4.2011. This daily pro-government newspaper published in the Kingdom of Bahrain is known for its interpretation of the events from the position of Arabic nationalism and is closely associated with the Prime Minister of Bahrain and the Egyptian Muslim Brotherhood. The newspaper provides a critical point of view denouncing the United States’ invasion of Iraq and Iraqis collaborating with the United States. It is a fellow newspaper to the English language daily, the Gulf Daily News. The outcomes presented in Figure 4 and Table 3 demonstrate the found change points.

1. December-2010
  - December 5, 2010
  Although a significant entropy drop characterized this point, we have not found any critical event connected to this date. We could suppose that this occurred due to internal changes in the newspaper’s management.
2. February-2011
  - February 17, 2011
  - February 23, 2011
  The presented group is obviously related to the protests that began on 14.2.2011. They led to a violent reaction from security forces with many victims within the Shia Muslim protesters.
  Uprisings extended to March with growing aggression from both sides. The Saudi-led intervention in Bahrain on 14.3.2011 was intended to support the Bahraini government in suppressing an anti-government revolt. The famous 16.3.20 crackdowns started from the capture of the government supporters, the University of Bahrain, and continued as a big media company aimed to chastise and discredit the protesting process. All these events are reflected by a change point arising in March 2011.
3. March-2011
  - March 25, 2011

#### 4.3.3. “Al-Ghad”

**Figure 5:**
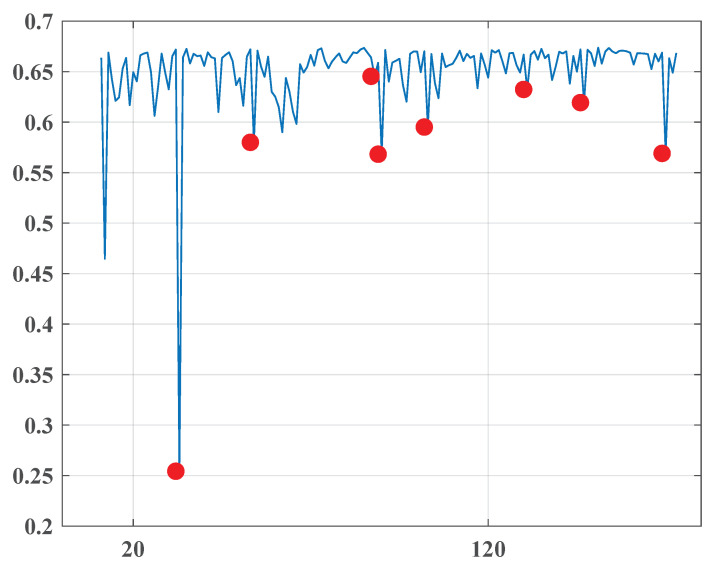
The overall entropy graph of the “Al-Ghad” newspaper.

This analyzed set included 83 issues of a Jordan newspaper “Al-Ghad” (The Tomorrow) collected in the period from 25.10.2010 to 25.4.2011. The following Figure 5 represents the detected change points together with Table 4.

Let us discuss these results.

1. November, December-2010
  - November 17, 2010
  - December 8, 2010
  There seemed to be a reaction to the 2010 Jordanian general election, which took place on 9.11.2010 behind the suspension of the previous parliament by King Abdullah II in November 2009. A wide range of parties boycotted the voting.
2. January-2011
  - January 11, 2011
  - January 13, 2011
  - January 26, 2011
  These dates correlated very well to the events of the Arab Spring in Jordan. The first demonstration was in Dhiban village on 7.1.2011 and was echoed in the capital city of Amman on 14.1.2011. On 28.1.2011, about 3500 protestors organized a demonstration with intense demands to improve significantly the economic situation in the State and make Prime Minister Samir Rifai step down.
3. March, April-2011
  - March 11, 2011
  - April 8, 2011
  Like the first group, it could be connected to the continuing protests in Jordan.

## 5. Conclusions and Discussion

The proposed method suggested a new dynamic pattern of the behavior of published traditional media. The model is based on the word embedding methodology and a statistical procedure intended to recognize significant changes in the social state via changes in the linguistic performers of the considered media. The provided experiments on several Arabic partially pro-government newspapers published in the Arab Spring age demonstrated that the approach is capable of detecting the notable changes mainly via compact groups of the discovered outliers. The authors suggest that a combination of the method with the item detection and sentiment analysis tools could adjust the approach’s performance and improve its ability to forecast the forthcoming events.

A likely additional aspect of the suggested method is applying as an indicator of the importance of happenings. This kind of methodology reverse usage could make it possible to rank events according to their impact on the media after the corresponding training of the model.

## Figures and Tables

**Table 1:** Change points detected for the “Al-Ahraam” newspaper in the first time frame.

1	October 27, 2010	0.608
2	November 17, 2010	0.593
3	January 11, 2011	0.665
4	January 13, 2011	0.637
5	January 26, 2011	0.644
6	February 23, 2011	0.66
7	March 11, 2011	0.654
8	April 3, 2011	0.637
9	April 24, 2011	0.658
10	May 2, 2011	0.652
11	May 18, 2011	0.512
12	June 21, 2011	0.661
13	June 28, 2011	0.66
14	June 30, 2011	0.657
15	August 7, 2011	0.664
16	August 21, 2011	0.665
17	October 2, 2011	0.667
18	October 14, 2011	0.667
19	October 23, 2011	0.653
20	November 24, 2011	0.654
21	November 29, 2011	0.655
22	December 6, 2011	0.641

**Table 2:** Change points detected for the “Al-Ahraam” newspaper in the second time frame.

1	January 11, 2014	0.559
2	January 12, 2014	0.103
3	March 16, 2014	0.609
4	April 11, 2014	0.629
5	May 5, 2014	0.646
6	May 11, 2014	0.639
7	May 20, 2014	0.561

**Table 3:** Change points detected for the “Akhbaar Al-Khaleej” newspaper.

1	December 5, 2010	0.31
2	January 8, 2011	0.623
3	January 11, 2011	0.627
4	January 18, 2011	0.62
5	January 21, 2011	0.601
6	February 17, 2011	0.537
7	February 23, 2011	0.509
8	March 25, 2011	0.591

**Table 4:** Change points detected for the “Al-Ghad” newspaper.

1	November 17, 2010	0.254
2	December 8, 2010	0.58
3	January 11, 2011	0.645
4	January 13, 2011	0.568
5	January 26, 2011	0.595
6	February 23, 2011	0.632
7	March 11, 2011	0.619
8	April 3, 2011	0.569
